# COVID-19-associated spontaneous subacute subdural haematoma: report of two cases

**DOI:** 10.1016/j.nmni.2021.100848

**Published:** 2021-02-12

**Authors:** A. Tabibkhooei, J. Hatam, M. Mokhtari, M. Abolmaali

**Affiliations:** 1) Department of Neurology, Rasoul Akram Hospital, Iran University of Medical Sciences, Tehran, Iran; 2) School of Medicine, Iran University of Medical Sciences, Tehran, Iran; 3) Shefa Neuroscience Research Centre, Khatam Alanbia Hospital, Tehran, Iran

**Keywords:** Coronavirus, coronavirus disease 2019, subdural haematoma

## Abstract

Since March 2020, the pandemic of coronavirus disease 2019 (COVID-19) has become a threat to global health. Several kinds of coronavirus-associated disorders, including vascular involvements with neurological symptoms, have been reported worldwide. Here, we describe two individuals with COVID-19 with no history of traumatic brain injury nor of vascular injuries, who developed spontaneous subdural haematoma in a subacute process. Both individuals became lethargic and unresponsive during admission in the intensive care unit. Both have undergone emergent craniotomy with acceptable outcome. The first patient improved significantly and was discharged a week after surgery. However, the second individual had no improvement on her consciousness and died 3 days after surgery. Haemorrhagic events, including subdural haematoma, can happen during COVID-19 infection with several possible mechanisms. Brain imaging and further neurological evaluation must be performed in any individuals with COVID-19 who show signs of alteration in their state of consciousness.

## Introduction

In late December 2019, the new term coronavirus disease 2019 (COVID-19), caused by severe acute respiratory syndrome coronavirus 2 (SARS-CoV-2) infection, was presented to the world. It was first detected in the city of Wuhan, China but has since spread globally. On 11 March 2020, the World Health Organization characterized the spread of COVID-19 as a pandemic [1]. COVID-19 causes a spectrum of symptoms, ranging from mild respiratory symptoms to fatal uncommon cerebrovascular manifestations. Acute haemorrhagic necrotizing encephalopathy, intracerebral haemorrhage and acute ischaemic stroke have been reported in association with COVID-19 [2,3]. Subacute subdural haematoma (SDH) is a venous derived intracranial haemorrhage that is mostly associated with traumatic brain injury. In this report, we present the incidence of spontaneous subacute SDH in two COVID-19-positive patients with few of the SDH risk factors.

## Case reports

### Case 1

**Fig. 1:**
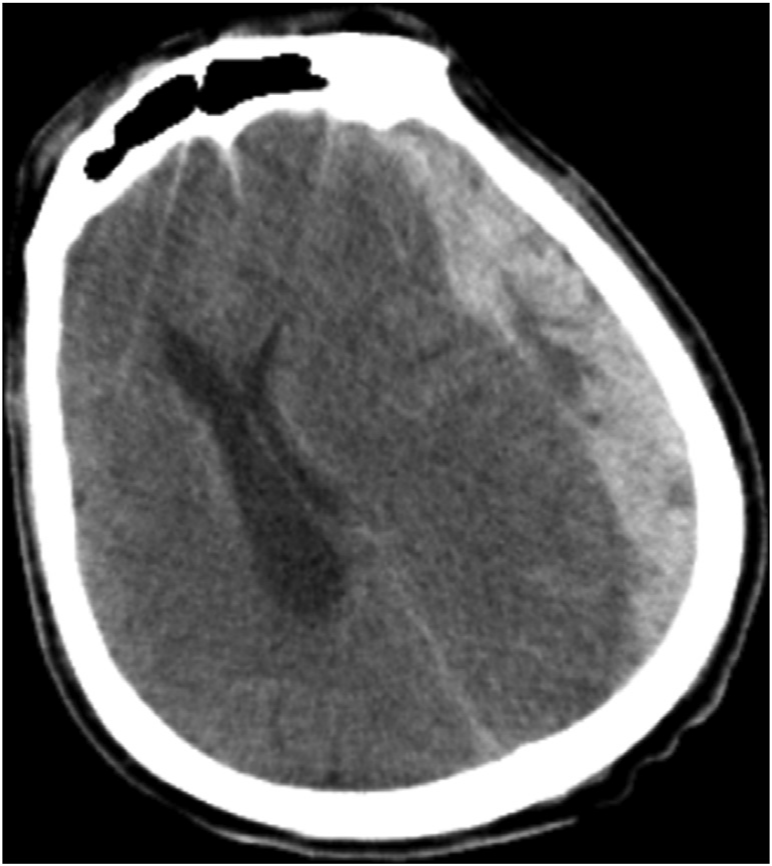
Brain CT scan, showing left fronto-temporo-parietal subacute haematoma with midline shift.

**Fig. 2:**
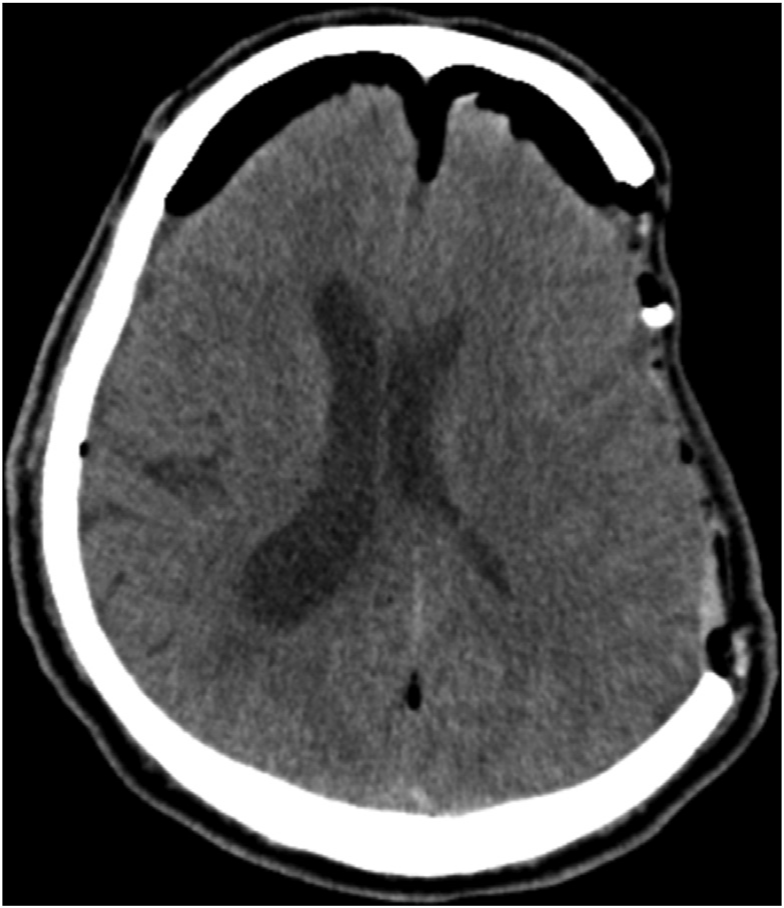
Post-operative brain CT scan, there was no haematoma or midline shift.

In early August 2020, A 69-year-old man was presented to the emergency department with complaint of dyspnoea, mild cough and fever. His symptoms began 10 days before admission. Past medical history was significant for diabetes mellitus, hypertension and ischaemic heart disease. He had been on metformin and losartan for over 7 years. Vital signs were as follows: blood pressure 115/75 mmHg, heart rate 84 beats/min, respiratory rate 25 breaths/min with oxygen saturation of 90% on room air and temperature 38.6°C. He had no history of loss of consciousness, falling or trauma. On general appearance, he was a fully alert and oriented older patient. Computed tomography (CT) of chest showed bilateral patchy ground-glass infiltration and was suggestive of SARS-CoV-2 infection. Nasopharyngeal COVID-19 PCR test returned positive, so he was isolated in the intensive care unit. He was commenced on dexamethasone along with hydroxychloroquine and lopinavir/ritonavir for COVID-19 treatment. On the third day of admission, the level of consciousness gradually declined. The patient became progressively unreactive to painful stimuli (Glasgow Coma Scale 4–5). Immediate brain CT imaging revealed a massive spontaneous subacute SDH on the left temporo-parietal region (Fig. 1). The patient was scheduled for an emergency evacuation of the haematoma via a left fronto-temporo-parietal craniotomy approach. Evacuation of haematoma (≈200 mL) and duraplasty were performed successfully with no postoperative bleeding (Fig. 2). The patient regained consciousness and was extubated a day after surgery. Postoperative days were uneventful. He returned to consciousness, the respiratory symptoms were resolved and he was discharged 7 days after the surgery with recommendation of subsequent quarantine.

### Case 2

**Fig. 3:**
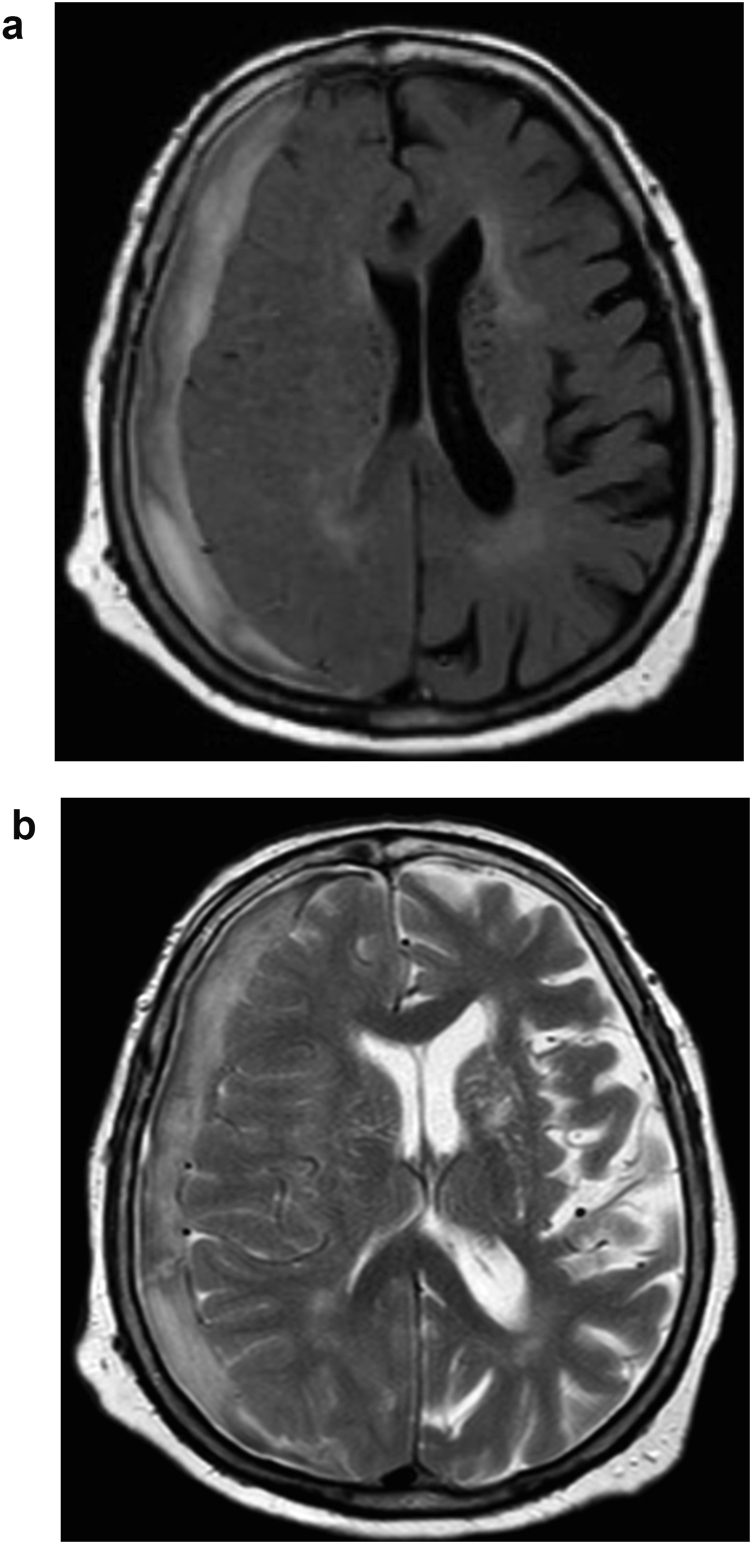
Brain MRI showing hyperintensity rim in T1W (a) and iso-to hypo-intensity in T2W (b) compatible with early subacute subdural haematoma.

**Fig. 4:**
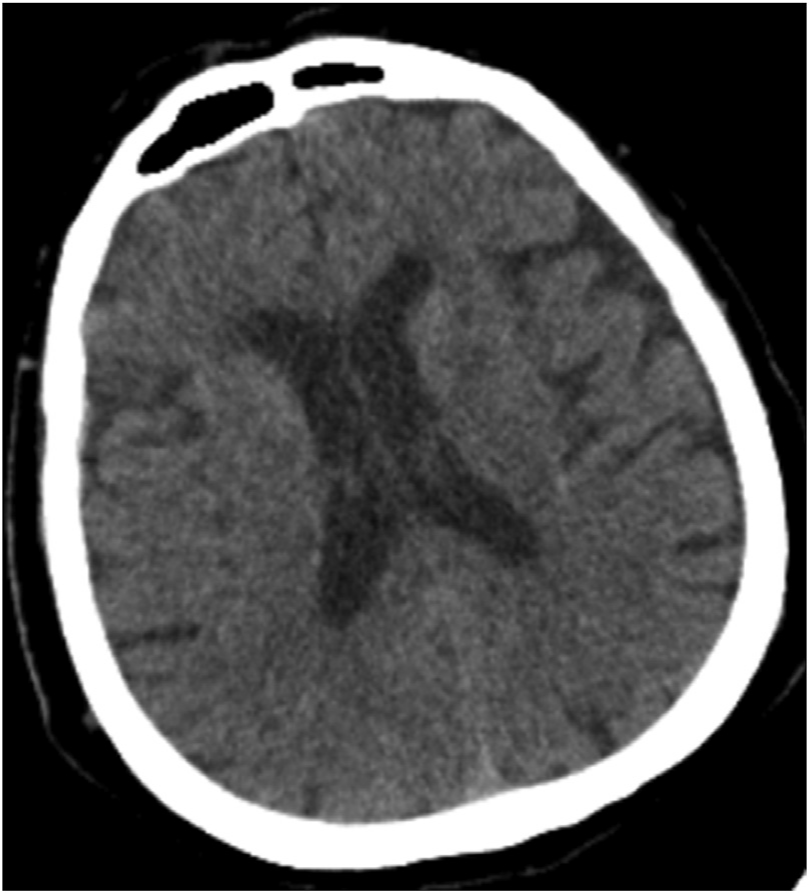
First admission day, brain CT scan showing no haematoma.

An 84-year-old woman was admitted to the emergency room in late August 2020 with respiratory symptoms. Her symptoms began 3 days before admission, with dry cough and progressive dyspnoea. She was afebrile and had no complaint of any other symptoms. Her medical history included hypertension. On physical examination, nothing significant was observed. Her oxygen saturation was 88%. She was obeying normally and was oriented to time, place, person and situation. Neurological examinations were also perfect. No history of decline in consciousness or trauma was observed in her history. She was admitted to hospital with an impression of COVID-19 infection. Chest CT scan was consistent with the diagnosis of COVID-19, which was then confirmed by a positive nasopharyngeal RT-PCR test. She was admitted to the intensive care unit for proper respiratory care and was treated with the suggested drugs for COVID-19 including remdesivir, hydroxychloroquine and lopinavir/ritonavir. The initial paraclinical findings revealed platelets of 270 × 10^6^/mL (normal range 140 × 10^6^–440 × 10^6^/mL), prothrombin time of 13 seconds (reference range 10–14 seconds), international normalized ratio of 1 and partial thromboplastin time of 44 seconds (reference range 24–40 seconds). During the second day of admission, the patient's level of consciousness deteriorated. An emergency consultation with the neurosurgery service was requested and brain magnetic resonance imaging (MRI) was performed,which revealed a subdural haemorrhage in the right fronto-temporo-parietal region (Fig. 3). The patient also had a brain CT scan on the day of admission on which no haemorrhage was visible (Fig. 4). An emergent right-sided craniotomy was performed and a volume of approximately 250 mL haematoma was evacuated from the subdural space. There was no improvement in her state of consciousness in the days post-surgery. Due to her critical condition, postoperative brain imaging was not possible and she died on the fifth day of admission.

## Discussion

With respect to the neurological manifestations of COVID-19, such as ischaemic stroke, myelitis, seizure, encephalitis and virus isolation from cerebrospinal fluid, it is understood that SARS-CoV-2 has a neurotropic and neuroinvasive nature [4]. The initial report from Rothstein *et al.* demonstrated the occurrence of ischaemic stroke, subarachnoid haemorrhage and intracerebral haemorrhage associated with SARS-CoV-2 in individuals with COVID-19, which was described as relatively uncommon [5]. Gogia *et al.* reported the first case of COVID-19-associated hyperacute SDH along with extensive intracerebral haemorrhage and subarachnoid haemorrhage in a 75-year-old patient. This individual was on double antiplatelet (aspirin and clopidogrel) treatment [6]; however, our cases had no history of antiplatelet or anticoagulant therapy. Different types of intracranial haemorrhage and their risk factors in patients with COVID-19 were described by Altschul *et al.* in November 2020 [7]. In their study, among 5227 individuals with COVID-19, 35 were found to have haemorrhage of some kind and 17 of the 35 had acute SDH. Based on the characteristics of these patients, 70.6% (*n* = 12) had a head trauma before the haemorrhage and five were on anticoagulant drugs [7]. However, such predisposing factors were not observed in our cases. Intracranial haemorrhage in individuals with COVID-19 were systematically assessed in a review by Cheruiyot *et al.* According to that study, out of 148 individuals with a diagnosis of intracranial haemorrhage, extracted from 23 studies, only 19 had a diagnosis of SDH and none of them were diagnosed with the subacute type of SDH [8].

Some mechanisms can be considered in the tendency of these patients to develop SDH. The point of entry for SARS-CoV-2 into human tissue is mediated primarily by a specific cellular receptor, angiotensin-converting enzyme 2 (ACE-2), which is expressed in various organs including brain parenchyma. In addition, ACE-2 receptors play an important role in vascular autoregulation and cerebral blood flow. Sharifi-Razavi *et al.* hypothesized that ACE-2 receptor dysfunction caused by direct invasion by SARS-CoV-2 may result in disruption of autoregulation and make the patient prone to vascular wall rupture in the presence of hypertension spikes [9]. In fact, systemic viraemia and subsequent endothelial dysfunction may make the bridging veins of the subdural space more vulnerable to bleeding following a minor trauma even to the point of sneezing, coughing or a Valsalva manoeuvre.

Vascular damage can also happen during viral infections that result in vasculitis, such as varicella zoster virus or human immunodeficiency virus [10].

Although the occurrence of thromboembolic events and a hypercoagulable state among patients with COVID-19 are well-described, haemorrhagic events should not be missed during the course of the disease. Several factors make patients susceptible to bleeding, including thrombocytopenia, hyperfibrinolytic state, excessive consumption of coagulation factors and receiving prophylactic anticoagulant agents [11]. Prolonged hypoxia of endothelial cells and pro-inflammatory state due to cytokine storm are the other possible causes that can result in endothelial damage and haemorrhage [3,12]. Increase in inflammatory markers such as matrix metalloproteinases along with increase in the level of tissue plasminogen activator may make the patient susceptible to haemorrhage into the subdural space with minor shear stress due to minor head trauma.

In conclusion, in addition to the thrombotic events that we expect in individuals with COVID-19, perhaps we need to pay more attention to haemorrhagic events, particularly in the brain. Acute decline of the level of consciousness is not necessarily tied to systemic conditions and the possibility of acute cerebrovascular haemorrhagic events must be kept in mind when treating patients with COVID-19.

## Declaration of competing interest

The authors declare that there is no conflict of interest.
